# Preparation and Characterization of Fenofibric Acid-Loaded Electrospun Fibrous Mats

**DOI:** 10.3390/nano16171111

**Published:** 2026-09-03

**Authors:** Enikő Bitay, Gergő Tóth, Zoltán-István Szabó

**Affiliations:** 1 Department of Mechanical Engineering, Faculty of Technical and Human Sciences, Sapientia Hungarian University of Transylvania, Calea Sighişoarei nr. 2, 540485 Targu Mures, Romania; 2 Bánki Donát Faculty of Mechanical and Safety Engineering, Óbuda University, Bécsi Street 96/b, 1034 Budapest, Hungary; 3 Research Institute of the Transylvanian Museum Society, 2–4 Napoca Street, 400009 Cluj-Napoca, Romania; 4 Department of Pharmaceutical Chemistry, Semmelweis University, Hőgyes Endre u. 9, 1092 Budapest, Hungary; 5 Center for Pharmacology and Drug Research & Development, Semmelweis University, 1092 Budapest, Hungary; 6 Department of Drugs Industry and Pharmaceutical Management, George Emil Palade University of Medicine, Pharmacy, Science, and Technology of Targu Mures, Gh. Marinescu 38, 540485 Targu Mures, Romania; 7 Sz-Imfidum Ltd., 525401 Lunga, Romania

**Keywords:** fenofibric acid, electrospinning, polyvinylpyrrolidone (PVP), microfibers, solubility enhancement

## Abstract

Fenofibric acid-loaded microfibrous mats were produced by electrospinning using polyvinylpyrrolidone (PVP) as a carrier polymer. Fenofibric acid, obtained through alkaline hydrolysis of fenofibrate, was incorporated into PVP solutions and processed under optimized electrospinning conditions to obtain uniform, defect-free fibers. The morphology and average fiber diameter were examined by scanning electron microscopy, revealing homogeneous, continuous fibers. Thermal analysis revealed the disappearance of the characteristic melting endotherm of fenofibric acid following electrospinning, indicating a substantial alteration in its thermal behavior within the polymer matrix. In vitro dissolution tests in artificial saliva showed a markedly enhanced release rate of fenofibric acid from the fibrous mats compared with the pure crystalline form, indicating a significant improvement in apparent solubility. These findings highlight the potential of PVP-based electrospun fiber formulation as an efficient carrier for the active metabolite of fenofibrate.

## 1. Introduction

Cardiovascular diseases (CVDs) remain among the leading causes of death worldwide, with dyslipidemia recognized as a key contributor to their onset and regression [1,2]. Increased plasma triglyceride and low-density lipoprotein (LDL) cholesterol levels accelerate atherogenesis and its complications. Among lipid-lowering therapies, fibrates occupy a prominent role due to their activation of peroxisome proliferator-activated receptor alpha (PPAR-α), a nuclear receptor that modulates lipid metabolism and promotes fatty acid oxidation [3].

**Figure 1:**
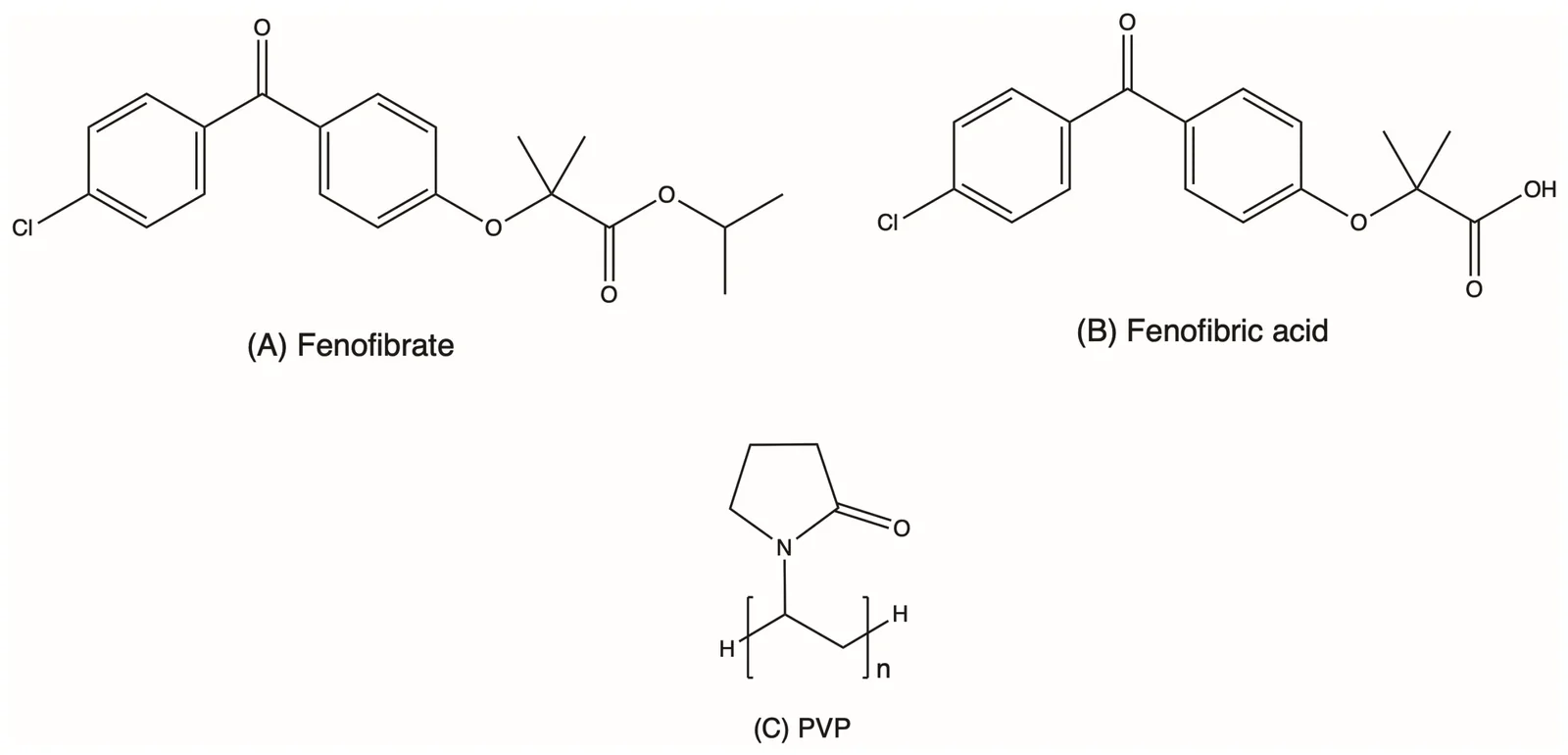
Chemical structures of (**A**) fenofibrate, (**B**) fenofibric acid, and (**C**) PVP.

Fenofibrate (Figure 1A), an isopropyl ester derivative of fenofibric acid (Figure 1B), has long been employed in the management of hyperlipidemia. After oral administration, fenofibrate undergoes rapid hydrolysis by esterases to yield fenofibric acid—the pharmacologically active moiety responsible for its lipid-lowering effect. As a potent PPAR-α agonist, fenofibric acid stimulates fatty acid β-οxidation, upregulating lipoprotein lipase activity, suppressing apolipoprotein C-III, and increasing the expression of apolipoproteins AI and AII, which decrease triglyceride and increase high-density lipoprotein (HDL) cholesterol levels. Furthermore, activation of PPAR-α reduces hepatic triglyceride synthesis and exerts anti-inflammatory and antioxidant effects in vascular tissues, contributing to its overall cardioprotective action [4,5].

Although fenofibrate has been extensively used, its pharmacokinetic profile is limited by poor aqueous solubility and variable bioavailability. It is classified as a Biopharmaceutics Classification System (BCS) Class II drug, characterized by high permeability but low solubility. Consequently, its oral absorption is limited by dissolution rate and strongly affected by food intake and interindividual variability in esterase activity [6,7]. Fenofibric acid exhibits improved solubility compared to fenofibrate and bypasses the need for enzymatic conversion; however, it still suffers from limited dissolution in physiological media and undergoes significant first-pass metabolism. These factors necessitate high oral doses and may lead to suboptimal therapeutic efficacy.

In recent years, polymer-based fibrous drug delivery systems have attracted considerable attention as an innovative approach to overcome the limitations of poorly water-soluble drugs [8,9,10]. Electrospun nanofibers provide a unique combination of large surface area, high porosity, and tunable fiber morphology, enabling enhanced dissolution rates and controlled drug release. Moreover, the ability to embed drugs in an amorphous or molecularly dispersed state within a polymer matrix significantly increases apparent solubility and dissolution kinetics [11,12].

The selection of an appropriate polymer is a key determinant of the structural and functional performance of fibrous mats. Biocompatible and biodegradable polymers such as polyvinyl alcohol (PVA), polycaprolactone (PCL), polyethylene oxide (PEO), and poly(lactic-co-glycolic acid) (PLGA) have been extensively employed in electrospinning for pharmaceutical applications [13,14,15,16,17]. Hydrophilic polymers like PVA, PEO, and PVP (Figure 1C) promote rapid matrix hydration and facilitate immediate drug release, while more hydrophobic polymers such as PCL and PLGA enable sustained or controlled release. By judiciously selecting and combining polymers, the release profile of fenofibric acid could be tailored to achieve desired therapeutic outcomes.

Owing to the poor aqueous solubility of fenofibric acid, improving its dissolution behavior represents an important formulation objective. Electrospun PVP-based fibrous systems are particularly attractive for this purpose, as the high specific surface area of the fibers, together with the potential reduction in drug crystallinity and the rapid dissolution of the hydrophilic polymer matrix, can substantially accelerate drug dissolution. 

In this study, electrospun PVP-based fibrous mats loaded with fenofibric acid were prepared and characterized. To the best of our knowledge, this is the first report describing the incorporation of fenofibric acid, the pharmacologically active metabolite of fenofibrate, into electrospun fibers. Unlike fenofibrate, which requires enzymatic hydrolysis to achieve therapeutic effect, fenofibric acid is administered in its pharmacologically active form. As it possesses distinct physicochemical properties, it also represents a different formulation target, unlike the ester prodrug. The objective was to develop a polymer-based fibrous system capable of enhancing the solubility and dissolution behavior of fenofibric acid through electrospinning and to perform its initial physicochemical characterization.

## 2. Materials and Methods

### 2.1. Materials

Fenofibrate was provided by a local pharmaceutical company from Târgu Mureș, Romania. 2-propanol, sodium hydroxide, hydrochloric acid (HCl) 32% (*w*/*w*), phosphoric acid 85% (*w*/*w*), ethyl acetate, acetonitrile, anhydrous sodium sulfate, disodium-hydrogen phosphate (Na_2_HPO_4_), potassium-dihydrogen phosphate (KH_2_PO_4_), and sodium chloride (NaCl) were acquired from Merck (Darmstadt, Germany). Ethanol and methanol were obtained from Chemical Company (Iași, Romania), while PVP (Plasdone K29/32) was obtained from ISP Technologies (Wayne, NJ, USA). Ultrapure water was prepared in-house, with the use of a Barnstead Nanopure Diamond water purification system (Boston, MA, USA).

Fenofibric acid was synthesized from fenofibrate through alkaline hydrolysis. In brief, 0.7 g of micronized fenofibrate was dissolved in 35 mL of chilled 2-propanol under magnetic stirring. After reaching room temperature, 0.77 mL of 50% sodium hydroxide solution was added, and the mixture was heated for three hours at 70–80 °C. The reaction mixture was diluted with distilled water, acidified to pH 2 with 1 M HCl, and extracted with ethyl acetate. The organic phase was dried over anhydrous sodium sulfate and filtered, and the organic solvent was evaporated at room temperature. The reaction was monitored using thin-layer chromatography on silica gel 60 RP-18, 5 × 10 cm, F254 plates (Merck, Darmstadt, Germany) using an acetonitrile–water mixture (16:4 *v*/*v*) as the mobile phase. The structure of the product was confirmed by 1H NMR spectroscopy and MS/MS. The results obtained (Supplementary Figures S1 and S2) are in accordance with those described in an earlier study [18].

### 2.2. Fiber Preparation by Electrospinning

Fibrous mats were prepared by electrospinning. The selected formulation was intended as a representative electrospun fenofibric acid system for physicochemical characterization. A viscous solution containing 4 g of polyvinylpyrrolidone (PVP K29/32) in 10 mL of ethanol was prepared, from which 1.7 g was withdrawn and supplemented with 0.1 g of fenofibric acid. After homogenization, the solution was transferred into a 2 mL syringe equipped with a 0.6 mm needle connected to a KDS 100 CE syringe pump (KD Scientific, Holliston, MA, USA) operating at 200 µL h^−1^. The collector, covered with aluminum foil, was positioned 12 cm from the needle, and a voltage of 25 kV was applied using an in-house built high-voltage power supply. Batches of fibrous mats were collected after 15 min of uninterrupted electrospinning.

### 2.3. Morphological Characterization of the Obtained Fibers

The formation of the fibers was first monitored using a Bresser LCD Micro 5 MP (Bresser GmbH, Rhede, Germany) optical microscope, and the obtained samples were subsequently also examined on a JSM 5200 (JEOL, Tokyo, Japan) scanning electron microscope (SEM) at a 15 kV accelerating voltage. Fiber diameters were determined from 20 measurements per SEM image at magnifications of ×1000 and ×5000 using ImageJ software (version 1.52a, National Institutes of Health, Bethesda, MD, USA).

### 2.4. Differential Scanning Calorimetric Studies

Differential scanning calorimetry (DSC) was performed on fenofibrate, fenofibric acid, physical mixture, placebo fibers, and fenofibric acid-loaded fibers. The apparatus employed for the thermoanalytical characterization was a Shimadzu DSC-60 (Shimadzu, Tokyo, Japan). Samples of 3–10 mg were placed into aluminum pans and sealed. An empty aluminum pan was used as reference. A single heat cycle was employed under air atmosphere in the temperature range of 30–250 °C, using a 10 °C/min heat rate.

### 2.5. Determination of Fenofibric Acid Content of the Fibers

Samples corresponding to approximately 10 mg of fenofibric acid-loaded fiber mats from three different batches were transferred into a 100 mL volumetric flask. Approximately 75 mL of methanol was added, and the obtained solutions were ultrasonicated for 5 min. After cooling, the solutions were completed to mark with methanol. A 1 mL amount of the obtained solution was further diluted to 10 mL with methanol. A standard solution of 10 μg/mL was also prepared in the same way. The prepared solutions were analyzed in a 10 mm quartz cuvette on a Shimadzu UV-1601PC UV-VIS spectrophotometer (Shimadzu, Tokyo, Japan) at 296 nm.

Loading efficiency of the fibers was determined using the following equation:

(1) $$Loading\;efficiency\left(\%\right)=\frac{C_{p}}{C_{t}}\times 100$$

where *C_p_* is the measured fenofibric acid concentration, and *C_t_* is the calculated (theoretical) fenofibric acid concentration of the fibers.

### 2.6. Small-Volume Dissolution Studies

Dissolution testing was conducted using an in-house-built setup described in our earlier publications. Briefly, 11.5 × 2.7 cm (height × ID) glass tubes were immersed in a water bath at 37 ± 1 °C, and temperature was maintained with the help of an Erweka ET 1500I immersion heather and thermostat (Erweka GmbH, Heusenstamm, Germany). Samples equivalent to 10 mg of fenofibric acid were dispersed in 30 mL of artificial saliva (2.38 g/L of Na_2_HPO_4_, 0.19 g/L of KH_2_PO_4_, and 8 g/L of NaCl; pH 6.8 adjusted with phosphoric acid). Stirring was performed at 300 rpm with the use of a magnetic stirrer (JK SMS HS stirrer, JKI, Shanghai, China) and Teflon-coated stir bars. Aliquots were withdrawn at 1, 3, 5, 10, 15, and 30 min, filtered through 10 µm Whatman filters (Cytiva, Buckinghamshire, UK), diluted to a 1:10 ratio with the dissolution medium, and analyzed spectrophotometrically at 296 nm. Fenofibric acid release was then calculated based on a calibration curve (Supplementary Figure S3).

## 3. Results and Discussion

### 3.1. Preparation and Morphological Characterization of Fenofibric Acid-Loaded Microfibers

**Figure 2:**
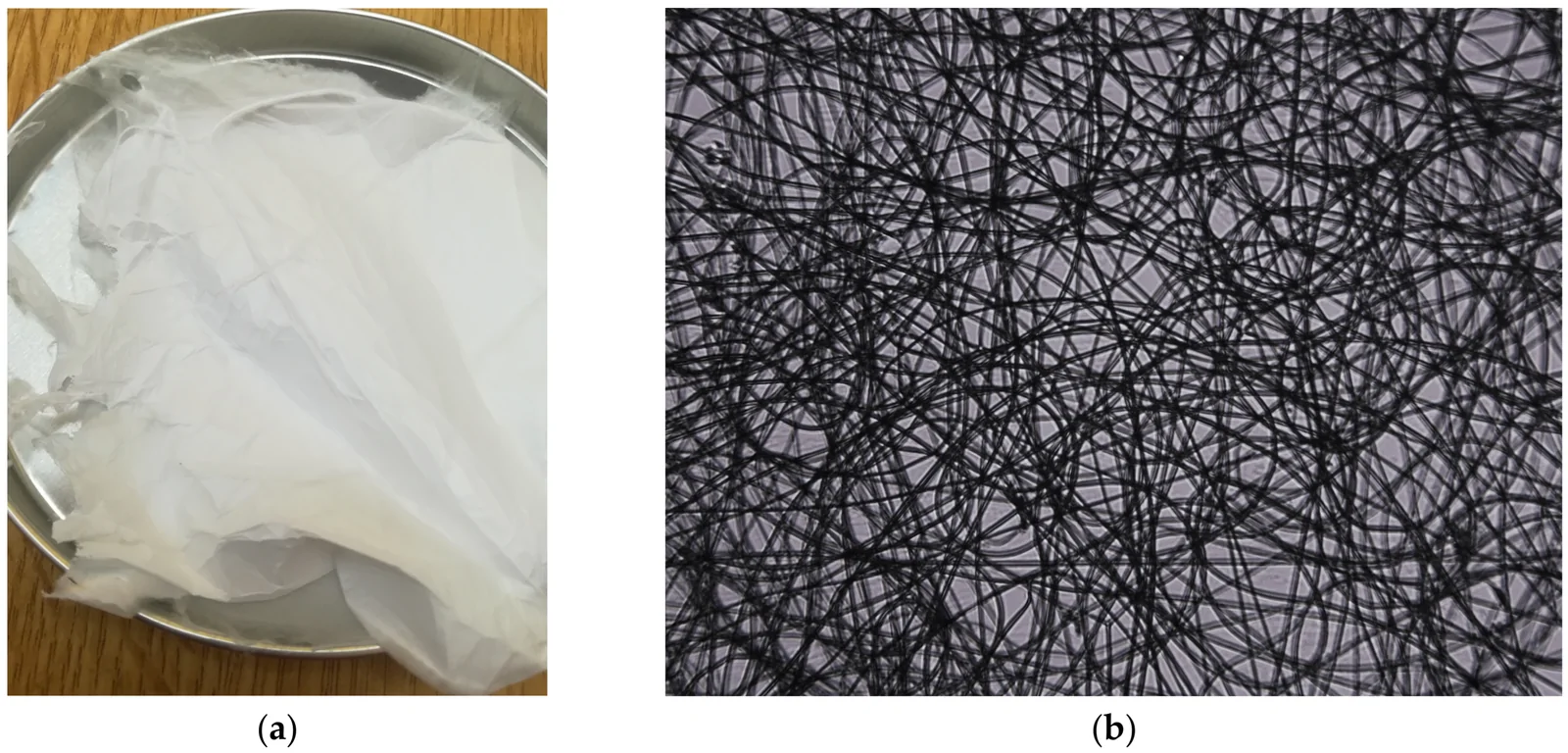
Macroscopic (**a**) and ×40 optical microscopic (**b**) images of the prepared fenofibric acid-loaded microfibrous mats.

**Figure 3:**
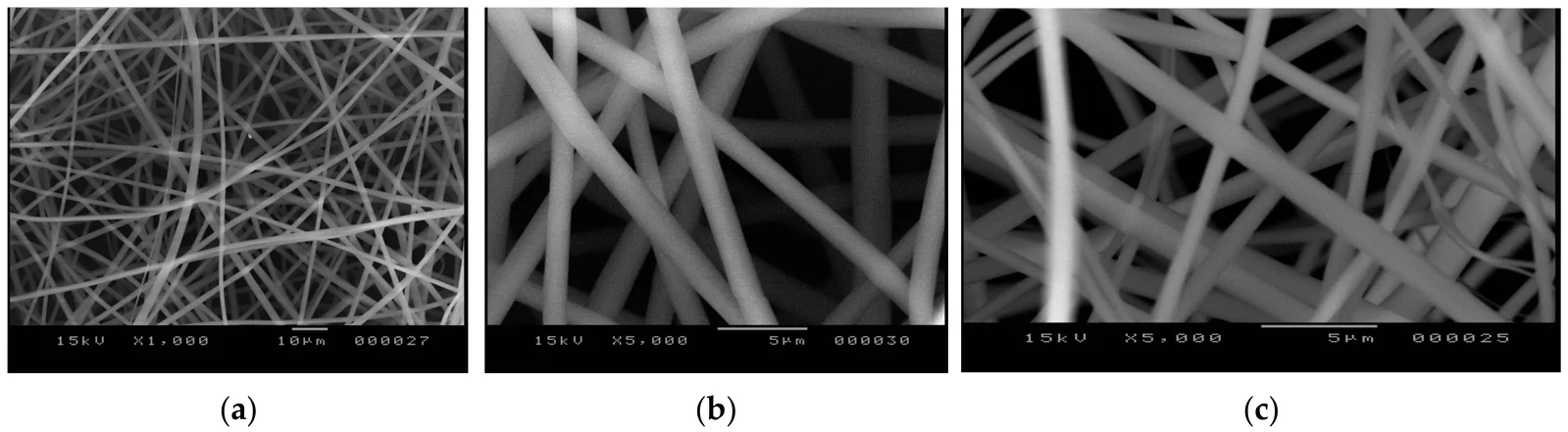
SEM micrographs of the obtained fenofibric acid-loaded microfibers at (**a**) ×1000 and (**b**) ×5000 magnifications; (**c**) placebo fibers at ×5000 magnification.

The electrospinning process yielded free-standing, self-supporting fibrous mats that could be readily removed from the collector. Representative macroscopic and optical microscopic images of the fenofibric acid-loaded fibrous mats are presented in Figure 2. To evaluate the influence of drug incorporation on fiber morphology, fibrous mats were prepared both in the presence and absence of fenofibric acid (placebo). In both formulations, defect-free, homogeneous, continuous fibers with smooth surfaces were obtained (Figure 3). The average fiber diameters were 779 ± 165 nm and 1150 ± 358 nm for the placebo and fenofibric acid-loaded formulations, respectively (histograms of fiber distribution are presented in Supplementary Figure S4). Although the viscosity of the electrospinning solutions was not measured, the observed increase in fiber diameter is consistent with the well-established relationship between increased solution viscosity and larger electrospun fiber diameters [19,20]. The obtained fiber morphology and average diameter are in line with earlier publications reporting microfibrous structure when ethanolic PVP solutions were used for electrospinning [21].

### 3.2. Thermoanalytical Characterization of the Obtained Microfibrous Mats

**Figure 4:**
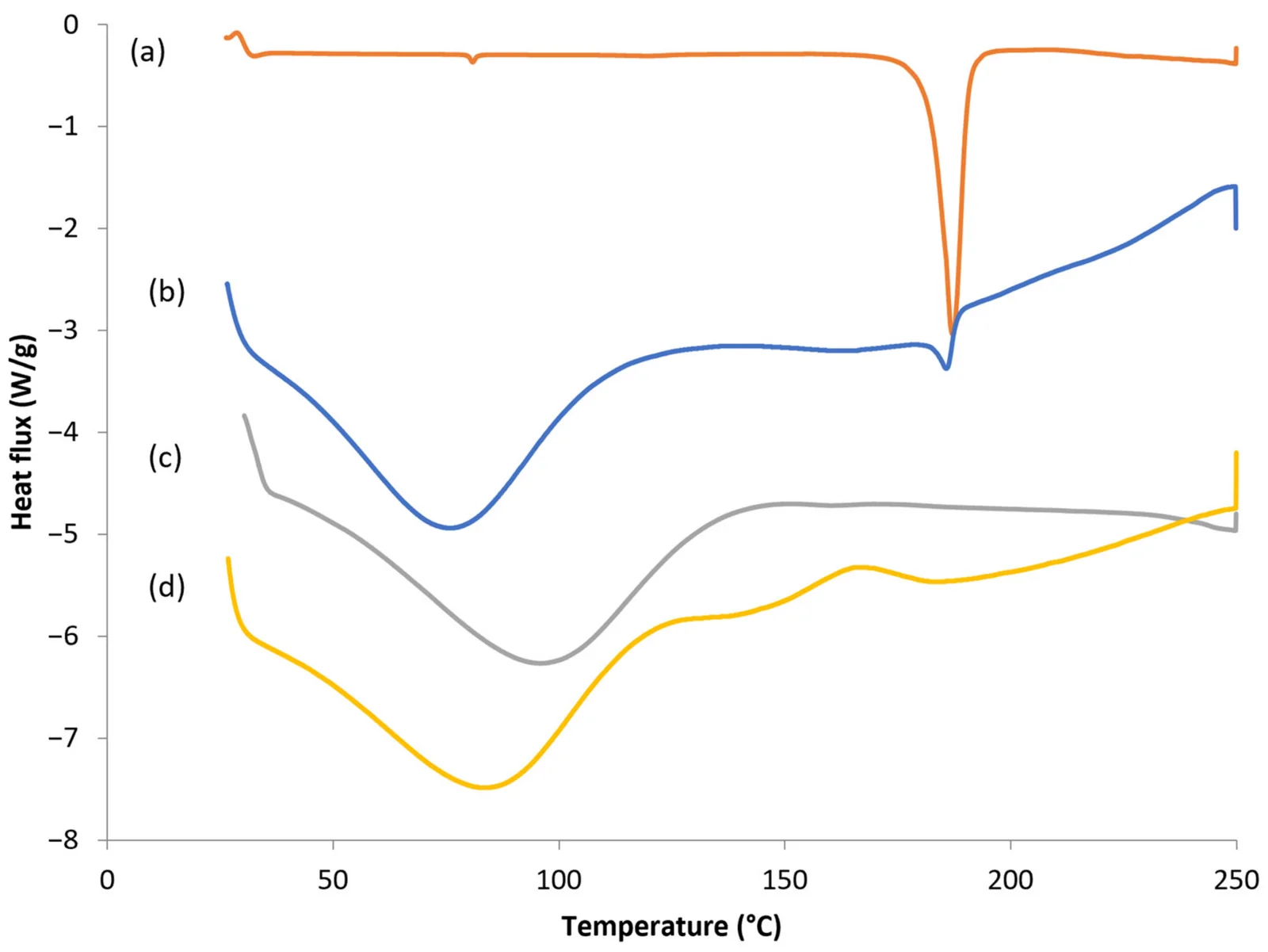
DSC thermograms obtained for (**a**) fenofibric acid, (**b**) physical mixture, (**c**) placebo fibers, and (**d**) fenofibric acid-loaded microfibers.

The DSC thermogram of the active substance, fenofibric acid (Figure 4a), displays a sharp endothermic peak around its characteristic melting point (≈187 °C), confirming the presence of the stable crystalline form of the active substance. This sharp transition serves as a reference for comparing the thermal behavior of the obtained fibrous systems. On the DSC curve of the physical mixture of PVP and fenofibric acid (Figure 3b), the characteristic melting endotherm of the active substance remains detectable; however, it appears reduced in intensity and slightly broadened. This attenuation of the peak is consistent with the lower proportion of the drug within the polymer matrix, resulting in a correspondingly weaker thermal response. The persistence of the melting transition nevertheless indicates that fenofibric acid retains its crystalline structure when combined with PVP solely through physical blending.

In the thermogram of the drug-loaded microfibers (Figure 4d), the characteristic melting endotherm of fenofibric acid is entirely absent, and only a broad dehydration event—arising from the hygroscopic nature of the fiber-forming polymer—is observed. A similar broad endothermic region is also present in the placebo fiber thermogram, confirming that this signal originates solely from the polymer and its associated moisture content. The characteristic melting endotherm of fenofibric acid was not detectable in the drug-loaded fibers, indicating a substantial alteration in its thermal behavior following electrospinning. This observation is consistent with a reduction in drug crystallinity; however, DSC alone does not allow the solid-state form of fenofibric acid within the fibers to be unequivocally established.

The observed change in thermal behavior may be related to the rapid solvent evaporation occurring during electrospinning, which can limit molecular organization and crystal formation. Nevertheless, the absence of the melting endotherm alone cannot unequivocally distinguish between reduced crystallinity and other possible solid-state arrangements of fenofibric acid within the PVP matrix.

Overall, the thermal analysis demonstrates a substantial change in the thermal behavior of fenofibric acid following electrospinning. The disappearance of the characteristic melting endotherm is consistent with reduced drug crystallinity, which may contribute, together with the hydrophilic PVP matrix and the high surface area of the fibers, to the improved dissolution behavior observed in subsequent experiments.

### 3.3. Determination of Fenofibric Acid Content in the Prepared Microfibers

The drug content of the electrospun microfibers was quantified using a UV–Vis spectrophotometric method, with measurements performed at 296 nm, corresponding to the absorbance maximum of fenofibric acid. The results presented in Table 1 indicate that the determined drug content ranged from 14.38% to 15.65%, representing 99.22–106.61% of the theoretical loading. Drug content analysis of three independently prepared electrospinning batches demonstrated good batch-to-batch reproducibility. These results suggest that the electrospinning process produced fibrous mats with consistent drug incorporation.

### 3.4. In Vitro Dissolution Studies

**Figure 5:**
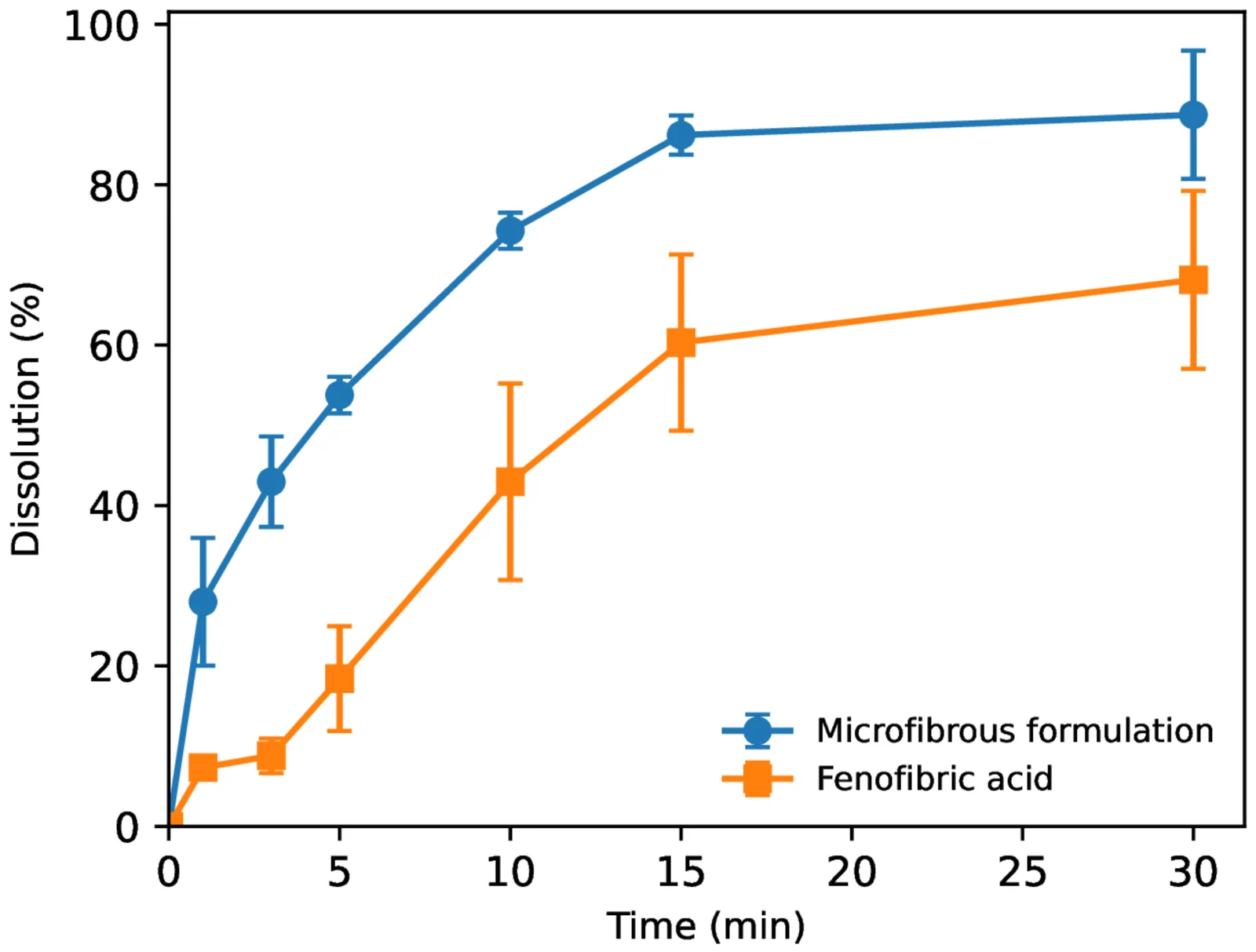
Results of comparative small-volume in vitro dissolution studies.

The comparative dissolution profiles of crystalline fenofibric acid and the electrospun microfibrous formulation are displayed in Figure 5.

The results indicate a clear enhancement in dissolution performance for the fibrous system. The microfibers exhibit a rapid initial release, with approximately 30% of the drug dissolved within the first minute, whereas the crystalline material dissolves only to a limited extent during the same period. This early-stage difference is suggested to be due to the substantially higher specific surface area and the enhanced wetting kinetics of electrospun structures compared with bulk crystalline solids.

The rapid dissolution of the PVP matrix upon contact with the dissolution medium was an intended design feature of the present formulation. PVP was selected as the carrier polymer to enable rapid hydration and disintegration of the fibrous network, thereby facilitating fast dissolution of the incorporated fenofibric acid. Colnsequently, the rapid loss of fiber integrity in aqueous media is consistent with the intended application of the system as an immediate-release formulation rather than a sustained-release drug delivery platform.

As dissolution progresses, the difference between the two systems becomes more apparent. After 15 min, more than 80% of the drug is dissolved from the microfibrous formulation, compared with approximately 60% from crystalline fenofibric acid. The accelerated dissolution of the microfibers may be related to several factors, including the hydrophilic nature of the PVP matrix, the high specific surface area of the fibrous structure, and the apparent reduction in drug crystallinity suggested by the DSC findings [22,23,24].

From 15 to 30 min, the dissolution of the microfibers progresses only slightly, approaching close to 90% by the end of the measurement, whereas the dissolution of crystalline fenofibric acid continues to increase gradually without reaching comparable levels. This behavior is consistent with the known solubility limitations of the crystalline drug. Similar trends have been described in the literature for electrospun systems containing poorly soluble compounds, where reduced crystallinity and incorporation into hydrophilic polymer matrices contribute to improved dissolution kinetics.

The dissolution profiles demonstrate that the microfibrous formulation offers a more favorable dissolution profile than crystalline fenofibric acid, with both faster release and a higher extent of dissolution. The rapid dissolution observed for the electrospun formulation is consistent with the intended design of the PVP-based fibrous system. The combination of the hydrophilic polymer matrix, the large specific surface area of the fibers, and the apparent reduction in drug crystallinity following electrospinning may contribute to the accelerated dissolution compared with crystalline fenofibric acid. These findings indicate that electrospinning represents a promising approach for enhancing the dissolution behavior of poorly water-soluble fenofibric acid. Nevertheless, additional studies are required to determine whether the improved in vitro dissolution translates into enhanced in vivo performance.

## 4. Conclusions

The present study demonstrates, to the best of our knowledge, the first successful preparation of electrospun PVP-based fibrous mats containing fenofibric acid, the pharmacologically active metabolite of fenofibrate. Electrospinning enabled the successful fabrication of uniform, defect-free microfibrous mats, providing the first physicochemical characterization of this formulation and demonstrating its potential as a rapidly dissolving fibrous drug delivery system. Thermal analysis confirmed the disappearance of the characteristic melting endotherm of the active substance in the fibrous samples, indicating a substantial alteration in its thermal behavior and suggesting a reduction in drug crystallinity following electrospinning. Spectrophotometric analysis confirmed that the microfibers contained the active pharmaceutical ingredient in a homogeneous and reproducible manner, with measured values closely matching the theoretical loading. The dissolution studies further revealed that the microfibrous formulation exhibits faster and more extensive drug release than the crystalline form. While the present work provides an initial physicochemical characterization of this formulation, complementary solid-state characterization, long-term stability studies, and in vivo evaluation will be required to fully assess the potential of the developed microfibrous system.

## Figures and Tables

**Table 1:** Drug content of the prepared microfibers determined by UV spectroscopy.

Sample	Fenofibric Acid Content of Fibers (%)	Loading Efficiency (%)
1	14.38	100.66
2	14.56	99.22
3	15.65	106.61
Mean	14.87	102.16
SD	0.69	3.92

## Data Availability

The data presented in this study are available upon request from the corresponding author.

## Supplementary Materials

The following supporting information can be downloaded at https://www.mdpi.com/article/10.3390/nano16171111/s1, Figure S1: Partial 1H NMR spectra illustrating the conversion of fenofibrate (top) to fenofibric acid (bottom). Hydrolysis of the isopropyl ester results in the complete disappearance of the characteristic isopropyl methine (δ ≈ 5.0 ppm) and methyl (δ ≈ 1.2 ppm) resonances, while the remaining signals are retained, confirming successful synthesis of fenofibric acid.; Figure S2: Comparative MS/MS product ion spectra obtained for fenofibrate and fenofibric acid in negative mode; Figure S3: Calibration curve used for the determination of fenofibric acid concentrations using UV spectroscopy; Figure S4: Histograms of fiber distribution of (A) placebo, and (B) fenofibric acid-loaded microfibers.
