# Supplementary material for: Preparation and Characterization of Fenofibric Acid-Loaded Electrospun Fibrous Mats
Source: Nanomaterials (Basel). 2026 Sep 3;16(17):1111. doi: 10.3390/nano16171111 (PMC13567033; doi:10.3390/nano16171111)
Supplement: Supplementary file 1 [file nanomaterials-16-01111-s001.zip › nanomaterials-4426755-supplementary.pdf]

Supplementary Material

Preparation and Characterization of Fenofibric Acid-Loaded Electrospun Fibrous Mats

Enikő Bitay [1,2,3], Gergő Tóth [4,5] and Zoltán-István Szabó [6,7,*]

1 Department of Mechanical Engineering, Faculty of Technical and Human Sciences, Sapientia Hungarian University of Transylvania, Calea Sighişoarei nr. 2, 540485 Targu Mures, Romania
2 Bánki Donát Faculty of Mechanical and Safety Engineering, Óbuda University, Bécsi Street 96/b, 1034 Budapest, Hungary
3 Research Institute of the Transylvanian Museum Society, 2–4 Napoca Street, 400009 Cluj-Napoca, Romania
4 Department of Pharmaceutical Chemistry, Semmelweis University, Hőgyes Endre u. 9, 1092 Budapest, Hungary
5 Center for Pharmacology and Drug Research & Development, Semmelweis University, 1092 Budapest, Hungary
6 Department of Drugs Industry and Pharmaceutical Management, George Emil Palade University of Medicine, Pharmacy, Science, and Technology of Targu Mures, Gh. Marinescu 38, 540485 Targu Mures, Romania
7 Sz-Imfidum Ltd., 525401 Lunga, Romania
* Correspondence: zoltan.szabo@umfst.ro

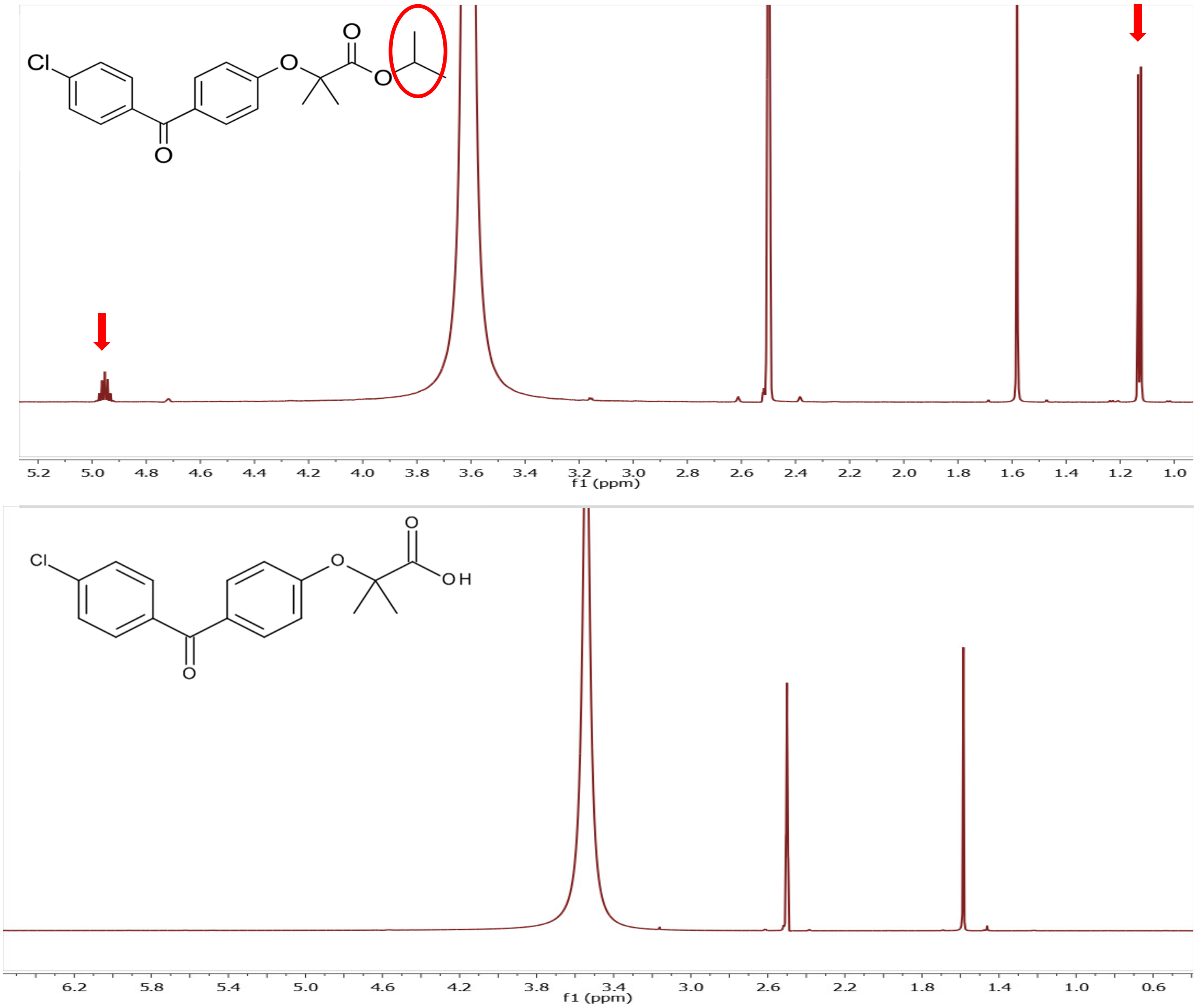

Figure S1. Partial 1H NMR spectra illustrating the conversion of fenofibrate (top) to fenofibric acid (bottom). Hydrolysis of the isopropyl ester results in the complete disappearance of the characteristic isopropyl methine (δ ≈ 5.0 ppm) and

methyl (δ ≈ 1.2 ppm) resonances, while the remaining signals are retained, confirming successful synthesis of fenofibric acid.

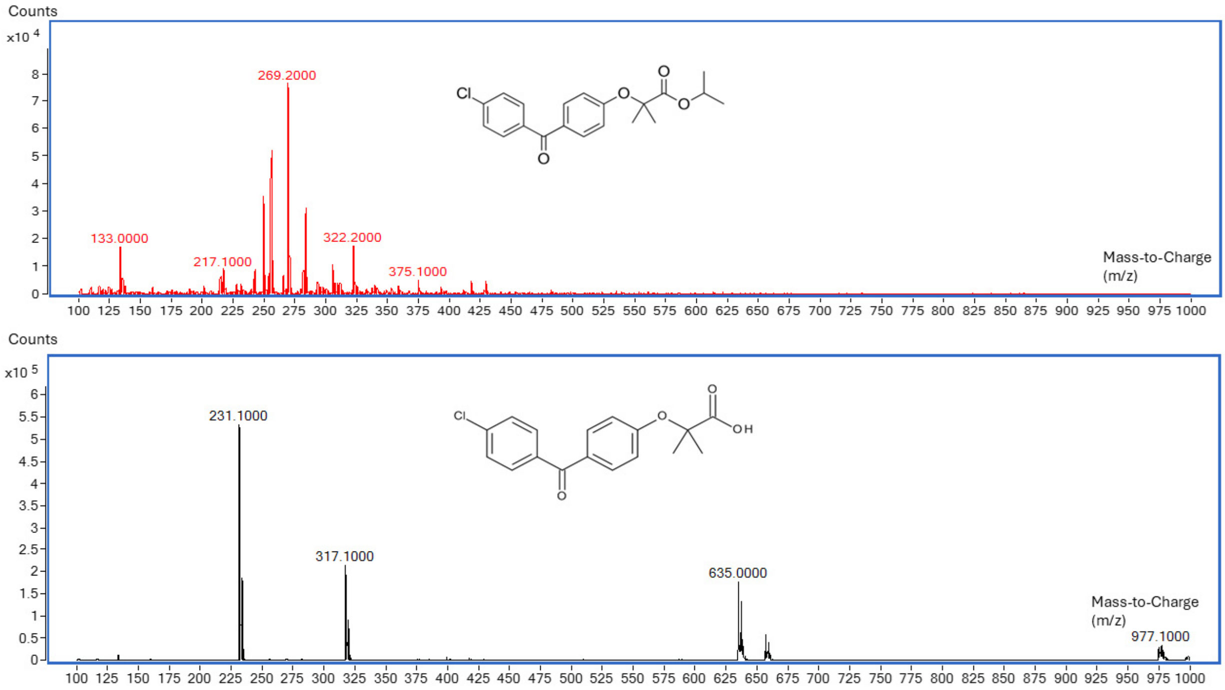

Figure S2. Comparative MS/MS product ion spectra obtained for fenofibrate and fenofibric acid in negative mode

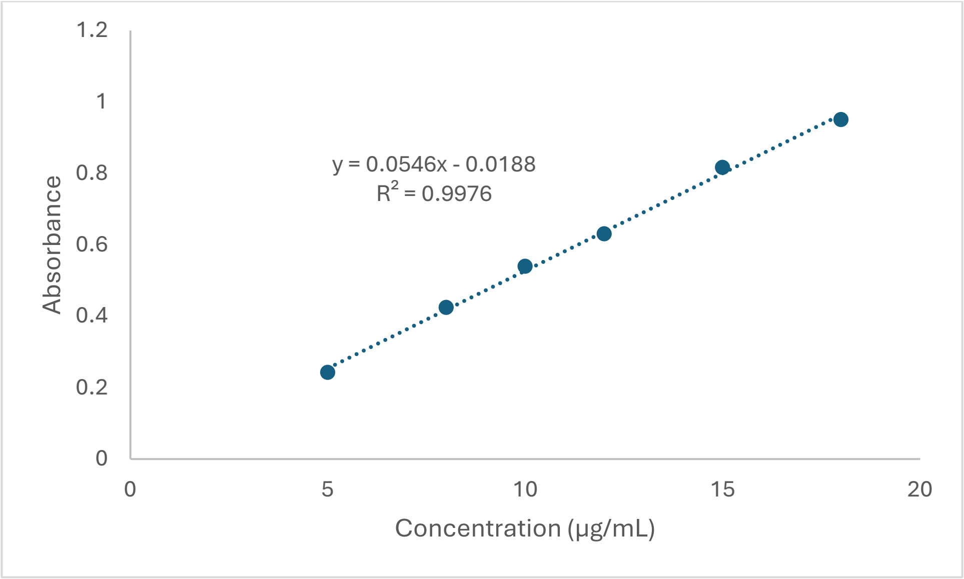

Figure S3. Calibration curve used for the determination of fenofibric acid concentrations using UV spectroscopy

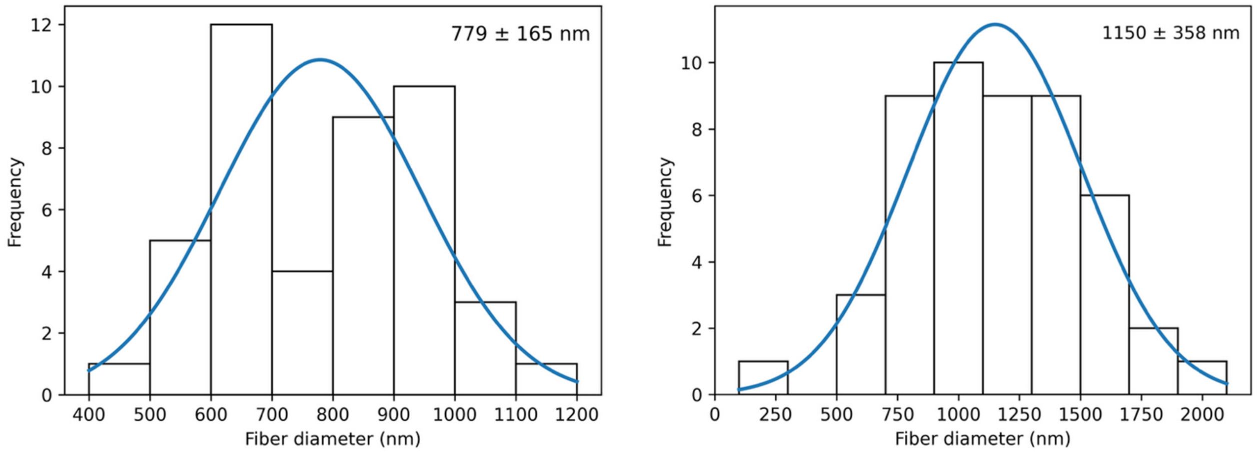

(A) (B)

Figure S4. Histograms of fiber distribution of (A) placebo, and (B) fenofibric acid-loaded microfibers
